# Combining epidemiological data and whole genome sequencing to understand SARS-CoV-2 transmission dynamics in a large tertiary care hospital during the first COVID-19 wave in The Netherlands focusing on healthcare workers

**DOI:** 10.1186/s13756-023-01247-7

**Published:** 2023-05-10

**Authors:** Cynthia P. Haanappel, Bas B. Oude Munnink, Reina S. Sikkema, Anne F. Voor in ’t holt, Herbert de Jager, Rieneke de Boever, Heidy H. H. T. Koene, Marjan Boter, Irina V. Chestakova, Anne van der Linden, Richard Molenkamp, Kara K. Osbak, Maris S. Arcilla, Margreet C. Vos, Marion P. G. Koopmans, Juliëtte A. Severin

**Affiliations:** 1 Department of Medical Microbiology and Infectious Diseases, Erasmus MC University Medical Center Rotterdam, 3000 CA Rotterdam, The Netherlands; 2 Department of Viroscience, Erasmus MC University Medical Center Rotterdam, Rotterdam, The Netherlands; 3 Department of Occupational Health Services, Erasmus MC University Medical Center Rotterdam, Rotterdam, The Netherlands

**Keywords:** COVID-19, SARS-CoV-2, Epidemiology, Nosocomial transmission, Hospital, Pandemic preparedness, Whole genome sequencing, Cluster analysis

## Abstract

**Background:**

Healthcare facilities have been challenged by the risk of SARS-CoV-2 transmission between healthcare workers (HCW) and patients. During the first wave of the COVID-19 pandemic, infections among HCW were observed, questioning infection prevention and control (IPC) measures implemented at that time.

**Aim:**

This study aimed to identify nosocomial transmission routes of SARS-CoV-2 between HCW and patients in a tertiary care hospital.

**Methods:**

All SARS-CoV-2 PCR positive HCW and patients identified between 1 March and 19 May 2020, were included in the analysis. Epidemiological data were collected from patient files and HCW contact tracing interviews. Whole genome sequences of SARS-CoV-2 were generated using Nanopore sequencing (WGS). Epidemiological clusters were identified, whereafter WGS and epidemiological data were combined for re-evaluation of epidemiological clusters and identification of potential transmission clusters. HCW infections were further classified into categories based on the likelihood that the infection was acquired via nosocomial transmission. Secondary cases were defined as COVID-19 cases in our hospital, part of a transmission cluster, of which the index case was either a patient or HCW from our hospital.

**Findings:**

The study population consisted of 293 HCW and 245 patients. Epidemiological data revealed 36 potential epidemiological clusters, with an estimated 222 (75.7%) HCW as secondary cases. WGS results were available for 195 HCW (88.2%) and 20 patients (12.8%) who belonged to an epidemiological cluster. Re-evaluation of the epidemiological clusters, with the available WGS data identified 31 transmission clusters with 65 (29.4%) HCW as secondary cases. Transmission clusters were all part of 18 (50.0%) previously determined epidemiological clusters, demonstrating that several larger outbreaks actually consisted, of several smaller transmission clusters. A total of 21 (7.2%) HCW infections were classified as from confirmed nosocomial, of which 18 were acquired from another HCW and 3 from a patient.

**Conclusion:**

The majority of SARS-CoV-2 infections among HCW could be attributed to community-acquired infection. Infections among HCW that could be classified as due to nosocomial transmission, were mainly caused by HCW-to-HCW transmission rather than patient-to-HCW transmission. It is important to recognize the uncertainties of cluster analyses based solely on epidemiological data.

## Background

The coronavirus disease 2019 (COVID-19) pandemic caused by severe acute respiratory syndrome coronavirus-2 (SARS-CoV-2) generated a significant burden on healthcare facilities worldwide [1]. Besides the large influx of COVID-19 patients, the nosocomial transmission of SARS-CoV-2 between patients and healthcare workers (HCW) has been a major concern.

Many studies have investigated SARS-CoV-2 outbreaks in healthcare facilities. However, many studies reporting on the nosocomial transmission of SARS-CoV-2 were in the context of either department-specific outbreaks or a select set of samples of the total SARS-CoV-2 positive population in a healthcare facility, whereby HCW data was not always available or analysed [2–5]. HCW experience community as well as occupational exposure to SARS-CoV-2, therefore HCW can play an important role in hospital outbreaks. It is important to determine what extent of COVID-19 among HCW is community- or hospital-acquired and how much they contribute to in-hospital transmission. Combining epidemiological and whole-genome sequencing (WGS) data can help elucidate the dynamics of SARS-CoV-2 hospital outbreaks, hereby allowing real-time adjustment of targeted infection prevention and control (IPC) measures. However, WGS is a technique not readily available for many healthcare facilities, especially not with a fast turn-around time. Consequently, many healthcare facilities rely on epidemiological data for their initial outbreak response. Therefore, it is necessary to investigate the over- or underestimation of outbreak clusters when using solely epidemiological data.

Here, we describe the transmission of SARS-CoV-2 between HCW and patients within a large tertiary hospital in The Netherlands during the first months of the COVID-19 pandemic. Secondly, we determine the added value of WGS in addition to epidemiological investigations with regard to outbreak investigation and source and contact tracing.

## Methods

### Setting

The Erasmus MC University Medical Center (Erasmus MC) is a large tertiary care hospital in Rotterdam, The Netherlands, with a total of 1100 beds and 39 operating rooms, including the Sophia Children’s Hospital. There are approximately 32,000 clinical admissions per year and 14,000 HCW employed (including physicians, registered nurses and researchers) [6]. The adult clinic primarily consists of single-occupancy rooms with private bathrooms, whereas the pediatric clinic mainly has multiple-occupancy rooms with shared bathrooms [7]. This analysis used data collected from 1 March 2020 until 19 May 2020 during the first wave of the COVID-19 pandemic. The end study date was chosen because no HCW tested positive for SARS-CoV-2 in the six weeks hereafter. At that time diagnostic test availability for SARS-CoV-2 was limited in The Netherlands; testing was only available to clinically suspected patients from COVID-19 risk groups, and hospital HCW. Until 19 May 2020 a total of 44,010 SARS-CoV-2 positive persons were registered in The Netherlands and 5,691 COVID-19 related deaths [8]. In the region of the hospital, Rotterdam-Rijnmond, a total of 4,252 COVID-19 cases were identified and 532 deaths in a population of 1.3 million inhabitants [8].

### Study design and data collection

All SARS-CoV-2 positive HCW and admitted patients, patients visiting the outpatient clinic, and patients visiting the emergency department who were tested between 1 March 2020 and 19 May 2020 were included. COVID-19 patients were either patients who tested SARS-CoV-2 positive upon admission (often referred by the general practitioner or transferred from other Dutch hospitals), at their hospital visit, or patients who tested positive during hospitalization. HCW and patients were tested for SARS-CoV-2 by reverse transcriptase-polymerase chain reaction (RT-PCR) on nasopharyngeal and throat swabs [9].

Epidemiological data collected from SARS-CoV-2 positive HCW included the date of positive SARS-CoV-2 test, age at the date of first positive SARS-CoV-2 PCR, date of symptom onset, symptom description, work location/department, job description, and self-reported source of infection. These data were prospectively collected as part of routine occupational health activity when the positive test result was shared with the HCW. Patient data extracted from electronic health records (EHR) included the date of positive SARS-CoV-2 PCR test, age at the date of the first positive SARS-CoV-2 test, hospital admission date, admission location, and previous contact with SARS-CoV-2 positive persons.

### Infection prevention and control measures

**Fig. 1:**
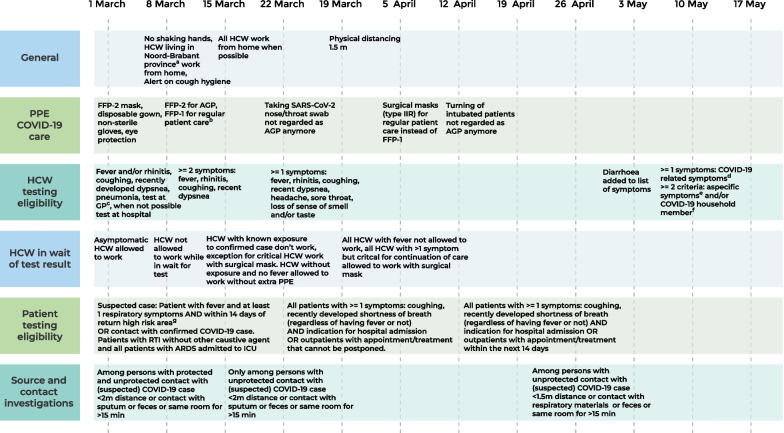
Timeline of infection prevention and control measures implemented in our hospital, per category. AGP = Aerosol generating procedures, ARDS = Acute respiratory distress syndrome, CI = Contact investigation, FFP = filtering face piece, GP = general practitioner, HCW = Healthcare workers, ICU = Intensive care unit, PPE = Personal protective equipment, RTI = Respiratory tract infection, m = meter. (a) Noord-Brabant is a neighboring province with a relatively high COVID-19 prevalence during the first wave. (b) Change from FFP-2 to FFP-1 masks in accordance to the national guideline at the time. (c) HCW who contacted their general practitioner for SARS-CoV-2 testing were referred to the municipal public health authorities. (d) Specific symptoms were: fever, coughing, shortness of breath, sore throat, and loss of sense of smell or taste. (e) Non-specific symptoms were: general malaise, fatigue, muscle ache, joint pain, gastrointestinal complaints and, pain behind the eyes. (f) Healthcare worker with a household member or partner with confirmed COVID-19 or with fever and respiratory symptoms. (g) China, Singapore, South Korea, Iran, Italy, Taiwan, Japan, Malaysia, Thailand, UAE, and Vietnam. From 10 March 2020 also the province of Noord-Brabant in The Netherlands

During the first months of the COVID-19 pandemic, national and Erasmus MC guidelines related to SARS-CoV-2 IPC measures were still being developed and adjusted according to new insights from experience and newly available literature. HCW did, however, always use personal protective equipment (PPE) when providing care for (suspected) COVID-19 patients. The recommended use of PPE changed over time, according to updated versions of national guidelines and new insights on transmissibility at the time. The Erasmus MC did not experience any shortage of PPE supply during the first COVID-19 wave. Details on the implemented IPC measures in the Erasmus MC during the study period are described in Fig. 1. For HCW, an occupational health facility for sampling and RT-PCR testing was available from 1 March 2020. Based on the routine occupational health information provided it was decided whether a source and contact investigation (CI) was necessary. These investigations were performed for each HCW that had been working with symptoms and for each patient that had not been cared for in adequate isolation conditions. Contacts were registered starting from the day of symptom onset.

### Whole genome sequencing and cluster identification

WGS was performed on all SARS-CoV-2 PCR positive HCW and patient nasopharyngeal swab samples with a cycle threshold (Ct) value of below 32. Nanopore sequencing was performed on these samples as described previously [10]. Successful sequencing was defined as having more than 90% genome coverage. The generated sequences and all other publicly available sequences from The Netherlands collected before 3 July 2020 were used for downstream analyses. Phylogenetic analysis was performed using IQ-TREE and trees were visualized using FigTree v.1.4.4 [11, 12]. Sequence clusters were identified as sequences from the same epidemiological cluster, same department and having a maximum of two nucleotide differences and sampled within two weeks [13]. Cluster definition for clusters between different departments was set on having a maximum of 1 nucleotide difference.

### Definitions

Epidemiological clusters were defined as two or more SARS-CoV-2 positive HCW or patients with a spatiotemporal link, either by unprotected contact < 14 days before symptom onset at the same hospital department, or known contact determined by source and contact investigations. Contact between patients and HCW whereby all required PPE and appropriate IPC measures were used were not regarded as transmission moments for the cluster analysis based on epidemiological data alone. When the date of symptom onset was not available the date of the first positive PCR test was used.

Transmission clusters were identified by re-evaluation of epidemiological clusters with the addition of WGS data. Hereby, transmission clusters were defined as a group of ≥ 2 SARS-CoV-2 positive HCW or patients with a link in time and place, confirmed by WGS data i.e. belonging to the same sequence cluster. This was done regardless of required PPE and appropriate IPC measures. Sequence clusters without epidemiological links were not regarded as transmission clusters.

For further classification of COVID-19 among HCW, we established definitions for the likelihood that nosocomial transmission had taken place (Table 1). Definitions were developed in a multidisciplinary group of epidemiologists, medical microbiologists and occupational health physicians, and were based upon literature and own experience [14]. For identification of an index case, it was assumed a minimum of 2 days and a maximum of 14 days between symptom onset of the index case and the secondary case was necessary to be able to appoint a definite index case. During instances where multiple indices of both HCW and patients were plausible, the index was classified as indeterminate. For classification a distinction was also made between regional and non-regional clusters. Regional clusters were defined as sequence clusters which were identified in ≥ 5 primary COVID-19 patients, whereby patients with a positive SARS-CoV-2 test upon admission were regarded as primary COVID-19 patients. We assumed primary COVID-19 patients were a good reflection of clusters circulating in the community. All other clusters were defined as non-regional.

**Table 1:** Definitions for the likelihood that healthcare worker infections were the result of nosocomial transmission

Likelihood		WGS result available	WGS cluster^a^	HCW and/or patient exposure^b^	Community exposure^b,c^
Confirmed transmission	**A**	SARS-CoV-2 PCR positive HCW with WGS result	Part of a non-regional sequence cluster	With exposure to other SARS-CoV-2 positive HCW and/or patients < 14 days before symptom onset	And without known exposure in the community
Probable transmission	**A**	SARS-CoV-2 PCR positive HCW with WGS result	Part of a **regional** sequence cluster	With exposure to other SARS-CoV-2 positive HCW and/or patients < 14 days before symptom onset	And without known exposure in the community
	**B**	SARS-CoV-2 PCR positive HCW with WGS result	Part of a non-regional sequence cluster	With exposure to other SARS-CoV-2 positive HCW and/or patients < 14 days of symptoms onset	And **with known exposure** in the community
Possible transmission	**A**	SARS-CoV-2 PCR positive HCW **without** WGS result		With exposure to other SARS-CoV-2 positive HCW and/or patients, **who were part of a WGS confirmed non-regional transmission cluster with ≥ 3 transmissions,** < 14 days before symptom onset	And without known exposure in the community
Transmission not confirmed	**A**	SARS-CoV-2 PCR positive HCW with WGS result	Part of a non-regional sequence cluster	But is the **index** case of the epidemiological cluster based on first date of symptom onset	And without known community exposure
	**B**	SARS-CoV-2 PCR positive HCW with WGS result	Part of a **regional** sequence cluster	With exposure to other SARS-CoV-2 positive HCW and/or patients < 14 days before symptom onset	And **with known exposure** in the community
	**C**	SARS-CoV-2 PCR positive HCW with WGS result	Part of a **regional or non-regional** sequence cluster or a **unique** viral strain	With exposure to other SARS-CoV-2 positive HCW and/or patients, **without WGS result**, < 14 days before symptom onset	And without known exposure in the community
	**D**	SARS-CoV-2 PCR positive HCW **without** WGS result		With known exposure to other SARS-CoV-2 positive HCW and/or patients < 14 days before symptom onset	
No transmission	**A**	SARS-CoV-2 PCR positive HCW with WGS result	Part of a non-regional sequence cluster	But is the **index** case of the epidemiological cluster based on first date of symptom onset	And **with known exposure** in the community
	**B**	SARS-CoV-2 PCR positive HCW with WGS result	Part of a **regional** sequence cluster	But is the **index** case of the epidemiological cluster based on first date of symptom onset	
	**C**	SARS-CoV-2 PCR positive HCW with WGS result	Part of a non-regional sequence cluster	But with **household member (HCW)** in the same epidemiological and genetic cluster	
	**D**	SARS-CoV-2 PCR positive HCW with WGS result	With a **unique** viral strain	And **without known exposure** to SARS-CoV-2 positive HCW and/or patients	
	**E**	SARS-CoV-2 PCR positive HCW **without** WGS result		And **without known** exposure to SARS-CoV-2 positive HCW and/or patients	

^a^Regional clusters were defined as sequence clusters which were identified in ≥ 5 primary COVID-19 patients, whereby patients with a positive SARS-CoV-2 test upon admission were regarded as primary COVID-19 patients. All other clusters were defined as non-regional

^b^Exposure to SARS-CoV-2 positive persons is defined as exposure < 14 days before symptom onset

^c^Community exposure is referred to as known exposure to SARS-CoV-2 infected individuals via household contacts, family, friends, events, and/or travels

HCW = healthcare worker, WGS = whole genome sequencing

### Data analyses

Epidemiological data were analyzed with SPSS version 28.0 (IBM, Armonk, NY, USA). Continuous variables were summarized as medians with range, and categorical variables were expressed as median numbers and percentages. For our retrospective cluster analysis, we first identified epidemiological clusters from all patients and HCW with a SARS-CoV-2 positive PCR. In a second analysis, the number and size of epidemiological clusters were determined only for patients and HCW with available WGS results. Thirdly, transmission clusters were established by combining epidemiological clusters and sequence clusters.

The number of secondary cases among HCW resulting from identified clusters was determined by subtracting the total number of index SARS-CoV-2 positive HCW from the total number of HCW in epidemiological or transmission clusters.

## Results

### Population characteristics: healthcare workers

**Fig. 2:**
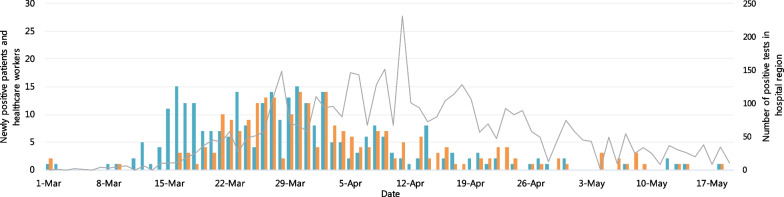
The number of newly positive SARS-CoV-2 tests per day. The number of newly SARS-CoV-2 positive patients and healthcare workers and the number of positive SARS-CoV-2 PCR tests in the hospital region per day. Blue = Healthcare workers; Orange = Patients; Grey = Region Rotterdam-Rijnmond

Between 1 March and 19 May 2020, 4362 HCW were tested for SARS-CoV-2 by RT-PCR at the Erasmus MC, of whom 293 HCW (6.7%) tested positive (Fig. 2). The  median age was 36 years (range 18–65) and 73% was female. Of positive HCW, 197 (67.2%) were clinical staff (e.g., nurses and physicians), of whom 11 (3.7%) HCW worked on a COVID-19 ward. The other 96 (32.8%) HCW did not work in direct patient care (e.g., administrative workers and analysts). The median time between symptom-onset and SARS-CoV-2 testing was 3 days (range 0–24 days).

Regarding self-reported sources of infection, 62 of 293 (21.2%) HCW reported a colleague, 33 (11.3%) HCW a patient, 28 (9.6%) HCW reported a family member, 21 (7.2%) HCW recent travel and 151 (51.5%) HCW did not report any possible source. Fourteen (4.8%) HCW reported multiple potential sources of infection.

Of HCW who tested positive, 162 out of 293 (55.3%) reported to have worked while symptomatic, whereby contact tracing was required for 103 (35.2%) HCW. For 56 HCW (19.1%) contact tracing among both HCW and patients was necessary, for 47 HCW (16.0%) only contact tracing among HCW was needed.

### Population characteristics: patients

During the study period, 245 patients tested positive. Patients had a median age of 62 years (range 3–94) and 35.7% were women. Out of 245 patients, 16 patients (6.5%) only had an outpatient visit while the other 229 patients (93.5%) were admitted as inpatients. Patients were admitted to the hospital for a median of 12.6 days (range 1–79 days). Contact tracing was required for 24 patients (9.8%).

### Epidemiological cluster analysis

**Fig. 3:**
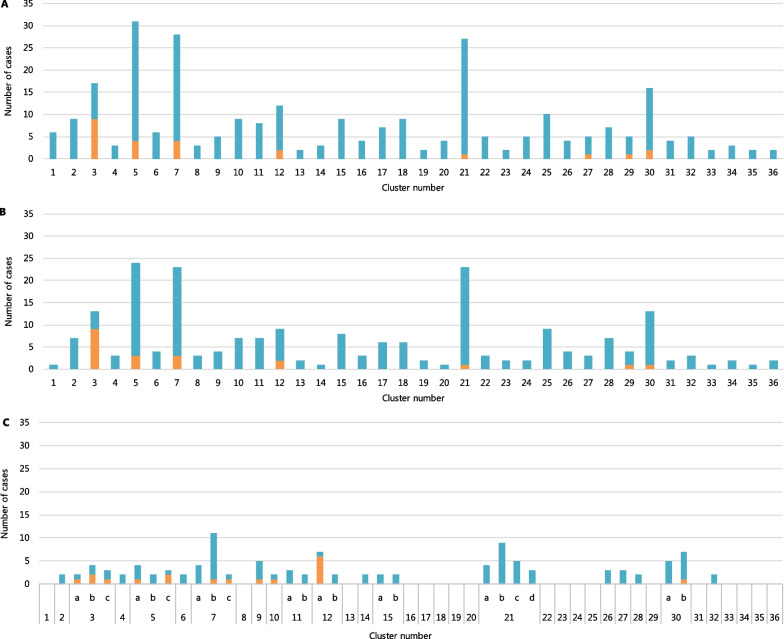
Identification of epidemiological clusters and transmission clusters. **A** Epidemiological clusters identified in the complete study population based only on epidemiological data. **B** Epidemiological clusters identified based only on epidemiological data, excluding cases without WGS results. **C** Transmission clusters identified in the study population confirmed by WGS. Epidemiological clusters portrayed in Fig. 3B were re-evaluated to form transmission clusters based on the combination of the epidemiological and sequence clusters. Different transmission cluster originating from the same epidemiological cluster are indicated with letters a/b/c/d. Blue = Healthcare workers; Orange = Patients

In total, 257 out of 293 HCW (87.7%) and 24 out of 245 patients (9.7%) were potentially part of an epidemiological cluster. Epidemiological data revealed 36 potential epidemiological clusters among the SARS-CoV-2 positive HCW and patients. Cluster size ranged from 2 to 31 cases and contained a median of 5 cases. Epidemiological clusters identified were found in 11 non-clinical departments, 15 inpatient departments, 7 outpatient departments, and 3 clusters in the operation room complex. Eight epidemiological clusters consisted of both patients and HCW, while the remaining 28 consisted of only HCW (Fig. 3A). Out of the 36 epidemiological clusters, only one cluster (cluster 3) had a patient as the index case, in all other clusters a HCW was the index case. Epidemiological cluster investigations resulted in the identification of 222 (75.7%) secondary cases out of 293 HCW.

### Sequencing and phylogenetic analysis

**Fig. 4:**
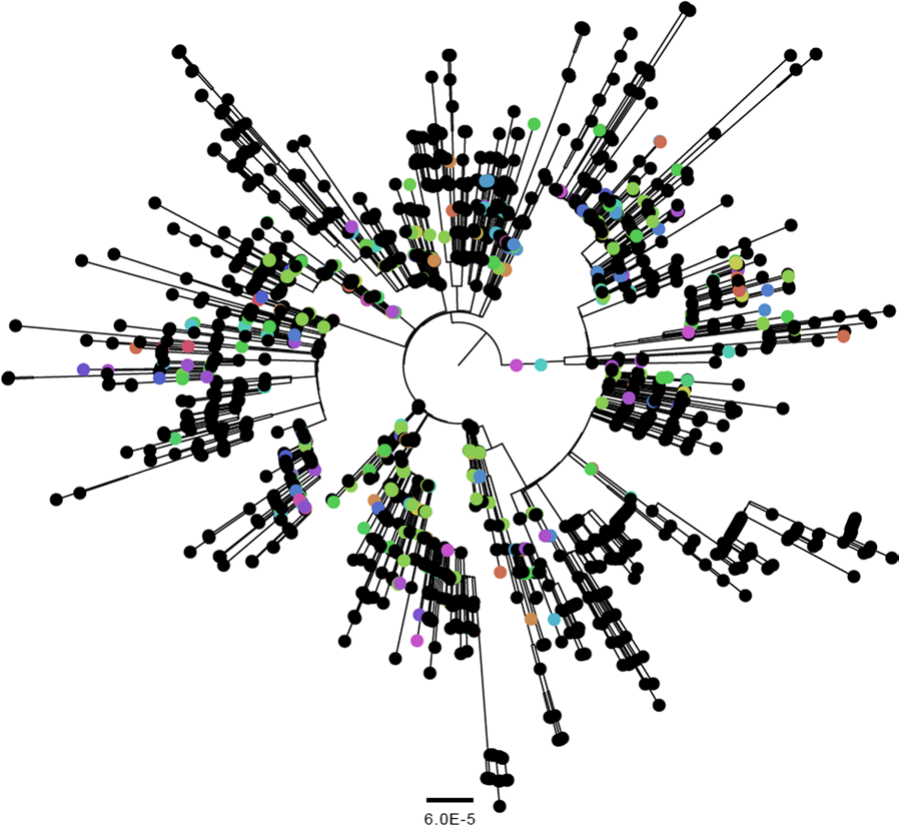
Phylogenetic analysis of all available sequences from the Netherlands on 3 July 2020. The different departments are depicted in different clusters. Thirty-five sequence clusters were identified, which made up 31 transmission clusters. The scale bar represents the number of nucleotide substitutions per site. Different colors represent different departments

WGS results were available for 195 HCW (88.2%) and 20 patients (12.8%) who belonged to an epidemiological cluster (Fig. 3B). Transmission clusters based on epidemiologic data combined with WGS contained a median of 3 persons (range 2–24). A total of 158 (71.5%) HCW were secondary cases. These sequences and all other sequences available from The Netherlands during the time of the study were used for phylogenetic analysis and cluster determination (Fig. 4).

A re-evaluation of the epidemiological clusters based on WGS data identified 31 transmission clusters (Fig. 3C). Five epidemiological clusters could not be further analyzed as there was only one person with available WGS data. The WGS determined 31 transmission clusters were part of 18 (50.0%) of the previously determined epidemiological clusters, demonstrating that several larger outbreaks actually consisted, of several smaller transmission clusters. These clusters consisted of 17 patients and 92 HCW. One hundred and thirty of 221 (58.9%) HCW did not belong to a cluster as they had a unique viral strain, indicating acquisition of the infection outside of the hospital. Eleven clusters consisted of both HCW and patients. Combining sequence and epidemiological clusters resulted in a total number of 65 HCW (29.4%) as secondary cases.

### Likelihood of nosocomial transmission among HCW

When SARS-CoV-2 positive HCW in transmission clusters were classified based on the likelihood of nosocomial SARS-CoV-2 transmission, the following was found: for 21 HCW (7.2% of all cases) there was confirmed nosocomial transmission, of which 18 acquired the infection from another HCW and 3 infections originated from a patient. For 37 HCW (12.6%) probable transmission was found and for 3 HCW (1.0%) possible transmission (Table 2). In five instances (1.7% of all cases) a patient was the most likely source for nosocomial transmission to a HCW. For the majority of the HCW (78.8% of all cases) the transmission could not be confirmed and/or were most probably infected outside the work setting.

**Table 2:** The likelihood that COVID-19 in a HCW was the result of nosocomial transmission

	HCW	
	N	%
Total	293	100
Confirmed transmission	21	7.2
Index HCW	18	
Index patient	3	
Index indeterminate	0	
Probable transmission	37	12.6
Index HCW	24	
Index patient	2	
Index indeterminate	11	
Possible transmission	3	1.0
Index HCW	2	
Index patient	0	
Index indeterminate	1	
Transmission not confirmed	78	26.6
No transmission	154	52.6

HCW = Healthcare worker. For all healthcare workers, including those without available whole genome sequencing data, the likelihood of nosocomial transmission was classified and the source of transmission was indicated

## Discussion

This comprehensive investigation of nosocomial SARS-CoV-2 transmission clusters revealed that the majority of SARS-CoV-2 infections among HCW could be attributed to community-acquired infection. Infections among HCW that could be classified as due to nosocomial transmission, were mainly caused by HCW-to-HCW transmission rather than patient-to-HCW transmission. Furthermore, we demonstrated that analyses based on epidemiological data alone largely overestimated the number of nosocomial transmissions, as well as the size of nosocomial transmission clusters.

SARS-CoV-2 has been widely recognized as an occupational health hazard for HCW [15, 16]. HCW have been shown to have a higher seroprevalence than the general population and a higher risk of severe COVID-19 [17, 18]. Especially during the initial phase of the pandemic, there were major concerns for nosocomial transmission of SARS-CoV-2 from patient-to-HCW. Because of these concerns, HCW testing was prioritized over community testing. Even though numerous IPC measures were in place, SARS-CoV-2 infections still occurred in HCW. Our analysis showed that only a very minor proportion of HCW infections (1.7%) were likely caused by patient-to-HCW transmission and limited nosocomial transmission took place from HCW-to-HCW. These results are in line with another Dutch study which identified multiple introductions of the virus among HCW through community-acquired infections [2]. Other studies reporting on hospital transmission dynamics have also pinpointed many different sources of infection for HCW outside the hospital, such as SARS-CoV-2 positive household members or contact with a potential case outside of work [17, 19, 20]. The findings of the study of Lindsey et al. similarly suggested that the majority of HCW were infected by another HCW [21]. In long-term care facilities, studies have shown that HCW posed a greater risk for patients rather than vice versa [22]. Contrary to our findings, Lumley et al. suggest that nosocomial transmission is underestimated [23]. This study however, focused on nosocomial acquisition of SARS-CoV-2 by inpatients rather than HCW and did not classify the likelihood of nosocomial transmission in HCW. Additionally, our setting with mainly single-occupancy rooms is different compared to settings with multiple occupancy rooms possibly resulting in different transmission dynamics.

A couple of explanations for HCW-to-HCW transmission can be listed; more than half of HCW (55%) reported working whilst symptomatic, reflected in the delay between the date of symptom onset and median test date three days later. Prior studies have noted that sickness presenteeism behavior among HCW is common for influenza-like illness and that HCW are known to be vectors for infectious diseases [24, 25]. Furthermore, the criteria for SARS-CoV-2 testing eligibility were quite stringent in our hospital in March 2020, partially due to the scarcity of tests and limited knowledge of the extent of COVID-19 symptoms. This could have contributed to the high number of HCW who remained working while symptomatic. When HCW in patient care were not working in patient rooms, for instance during coffee breaks or small meetings, masks were often not worn as universal masking was not implemented at our hospital during the first wave. Physical distancing and universal masking of HCW are IPC measures that can be implemented to assure fewer transmission events can take place [26, 27]. Masking should also be accompanied by proper hand hygiene and adequate doffing and donning [28]. Physical distancing was implemented in our hospital at the end of March 2020, however, implementation in practice took time. As HCW are essential workers, especially during a pandemic response, preventive measures for HCW-to-HCW transmission are important in addition to measures during contact with patients.

The comparison of cluster analyses demonstrated that identification of secondary cases through epidemiological data alone can result in substantial overestimation. These findings highlight once more the importance of investigating potential nosocomial transmission through a combination of detailed epidemiological investigation combined with WGS data [14, 21, 29]. Knowing the extent of overestimation of nosocomial transmission will help us understand and put the findings of epidemiologic outbreak investigations into perspective. One of the studies which clearly presented both epidemiological clusters and clusters with combined epidemiological and WGS data, is the study of Watt et al. [30]. Contrary to our findings, this study identified more nosocomial transmission compared to classical epidemiology using WGS data [30]. This discrepancy may be due to the extent of epidemiological data available for the initial epidemiological cluster analysis, difference in genomic cluster definition and difference in the community prevalence of SARS-CoV-2 during the study period.

### Strengths and limitations

Results of our study were obtained through a retrospective in-depth analysis combining epidemiologic and WGS data. However, for IPC and outbreak management WGS is often not readily available, requiring decision-making of real-time outbreak interventions to rely on epidemiological data alone. While many studies have previously highlighted the added value of WGS for in-depth cluster analysis, fewer studies have presented the disparity in results after adding WGS to the cluster analyses [30–32]. Factors which distinguish our study from others are our inclusion of the full (SARS-CoV-2 positive) hospital patient and HCW population and the provision of definitions on the likelihood of nosocomial transmission among HCW. Multiple outbreak investigations combining epidemiological and WGS data have been described, however, the majority of studies focus on hospital-acquired COVID-19 among patients rather than HCW. Therefore, studies often only describe definitions for hospital-acquired COVID-19 for patients and exclude these definitions for HCW, making it unclear what proportion of SARS-CoV-2 positive HCW is attributable to community transmission [26]. Studies that focus on SARS-CoV-2 transmission to HCW often describe risk factors for a positive SARS-CoV-2 PCR among HCW, but do not classify the likelihood of nosocomial transmission among HCW, nor pinpoint the actual number of SARS-CoV-2 positive HCW that can be attributed to nosocomial transmission [33].

Limitations of this study include missing WGS data due to low viral loads, which could have resulted in missing links. Another challenge in identifying and confirming nosocomial transmission is the relatively low genetic diversity of SARS-CoV-2 strains [22]. This can affect the cluster analysis in a way that separate community introductions or nosocomial transmission are indistinguishable based on WGS data. We regarded these cases as possible transmission and more information via detailed epidemiological data was crucial for the interpretation of the WGS data in outbreak investigation. By classifying the likelihood of nosocomial transmission among HCW, this factor of uncertainty due to regional clusters should be taken into account. This is especially true in the beginning of a pandemic when testing and subsequent sequencing of positive cases is biased towards high-risk groups (i.e., HCW) and hospitalized patients, and community surveillance is not yet performed, possibly leading to an overestimation of the identified clusters. Our study only comprises data from the first COVID-19 wave in 2020, and different factors have changed during the course of time such as SARS-CoV-2 variants, immune status and differences in community prevalence. However, the results of this study still highlight the challenges of pandemic preparedness and outbreak investigations when a new virus emerges.

Moreover, information regarding SARS-CoV-2 positive visitors was not registered and asymptomatic HCW and patients were not tested and therefore could not be taken into account. Up to 33% of SARS-CoV-2 infections in adults are estimated to be asymptomatic, therefore this could have resulted in missing links and clusters [34]. Only a fraction of all regional COVID-19 cases were tested and sequenced. This might have resulted in an underestimation of sequence diversity in the community and thus, regional clusters. Additionally, regional clusters were defined as having ≥ 5 primary patients, which is an arbitrary cut-off.

## Conclusion

The findings of this study highlight the contribution of SARS-CoV-2 community-acquired infections in HCW settings, the limited number of patient-to-HCW transmissions as well as the added value of WGS to epidemiological data. The COVID-19 pandemic has emphasized the importance of real-time outbreak management for pandemic preparedness. While epidemiological data such as source and contact tracing is important in hospital outbreak management and investigation, it may not suffice in scenarios of high community prevalence. Since WGS is not readily available for many healthcare facilities it is important to recognize the uncertainties of cluster analyses based solely on epidemiological data as well as to recognize the contribution of HCW-to-HCW transmission. The collaboration between the IPC team and occupational health services, together with the use of complementary techniques like epidemiological cluster analysis and WGS is essential to provide knowledge on nosocomial SARS-CoV-2 transmission dynamics. During this first wave of the pandemic, HCW testing was prioritized over community testing. Our study shows the importance of surveillance in the community in order to understand sequence clusters. Our study population originated from a single tertiary care center with single occupancy rooms, which could result in different transmission dynamics compared to healthcare facilities with multiple occupancy rooms as more contact occurs between patients. Future studies should investigate this difference.

## Data Availability

The datasets used and analysed during the current study are available from the corresponding author on reasonable request.

## Abbreviations

AGP: Aerosol generating procedures
ARDS: Acute respiratory distress syndrome
CI: Contact investigation
COVID-19: Coronavirus disease 2019
Ct: Cycle threshold
EHR: Electronic health record
Erasmus MC: Erasmus MC University Medical Center
GP: General practitioner
HCW: Healthcare worker
ICU: Intensive care unit
IPC: Infection prevention and control
M: Meter
NA: Not applicable
PPE: Personal protective equipment
RTI: Respiratory tract infection
RT-PCR: Reverse transcriptase-polymerase chain reaction
SARS-CoV-2: Severe acute respiratory syndrome coronavirus-2
WGS: Whole genome sequencing
